# TLR3 Agonist Amplifies the Anti-Inflammatory Potency of ADSCs via IL-10-Mediated Macrophage Polarization in Acute Pancreatitis

**DOI:** 10.1155/2024/5579228

**Published:** 2024-03-21

**Authors:** Jianxing Liu, Wenjing Yan, Shanshan Chen, Yingjie Sun, Fangfang Zhang, Yue Yang, Liang Jin

**Affiliations:** State Key Laboratory of Natural Medicines, Jiangsu Key Laboratory of Druggability of Biopharmaceuticals, School of Life Science and Technology, China Pharmaceutical University, 24 Tongjiaxiang, Nanjing, China

## Abstract

The immunoregulatory role of mesenchymal stem cells (MSCs) in inflammation is heterogeneous and can exhibit anti-inflammatory or proinflammatory properties depending on the microenvironment. We herein observed that the activation of Toll-like receptor 3 (TLR3) by polyinosinic : polycytidylic acid (poly(I : C)) stimulation facilitated the transformation of adipose-derived stem cells (ADSCs) into an anti-inflammatory phenotype. The enhanced anti-inflammatory properties were assessed in a taurocholate-induced pancreatitis model. The results demonstrated that poly(I : C) pretreated ADSCs exhibited enhanced anti-inflammatory properties than untreated ADSCs in taurocholate-induced pancreatitis. Mechanistically, poly(I : C)-treated ADSCs showed increased production and secretion of interleukin-10 (IL-10), which demonstrates a potent ability to alleviate inflammatory signaling cascades in acinar cells. Simultaneously, the heightened anti-inflammatory effects of poly(I : C)-treated ADSCs in pancreatitis were associated with the regulation of macrophage classical/alternative transformation, thereby mitigating inflammatory factor-mediated damage to the pancreatic acinar cell. We propose that TLR3 activation by poly(I : C) is an effective strategy to enhance the anti-inflammatory properties of MSCs, which offers a valuable consideration for improving the therapeutic efficacy of MSCs in inflammatory diseases.

## 1. Introduction

Mesenchymal stem cells (MSCs) have demonstrated extensive immunoregulatory capabilities, influencing both adaptive and innate immune responses [1]. Recent findings highlight MSCs' ability to actively sense pathogen-associated molecular patterns or damage-associated molecular patterns via Toll-like receptors (TLRs) on their surface [2], and these interactions play a pivotal role in modulating inflammation [3, 4]. In response to diverse pathogenic microenvironments, MSCs can transform into either a proinflammatory or anti-inflammatory phenotype (MSC1 and MSC2) following specific TLR or cytokine stimulation [5], resulting in distinct secretomes [6]. Specifically, TLR4-primed MSCs tend to adopt a proinflammatory profile (MSC1) characterized by elevated levels of interleukin (IL)-6 and IL-8 secretion. In contrast, TLR3-primed MSCs favor an anti-inflammatory profile (MSC2) marked by increased expression of indoleamine-2,3-dioxygenase (IDO) and prostaglandin E2 (PGE2) [7]. The difference between the anti-inflammatory and proinflammatory phenotypes of MSCs is not only represented in their differential secretory profiles but also in the procession of regulating other immune cells. Overall, MSC2 cells secrete high amounts of IDO and anti-inflammatory cytokines (TGF-*β* and IL-10) and thereby inhibit the activity of macrophages, neutrophils, and lymphocytes [8]. By contrast, MSC1 therapy may aggravate macrophage inflammatory cytokines production and promote the early or mild phase of peripheral inflammation than conventional MSC or MSC2 therapy [9]. The adaptable nature of MSCs in response to TLR activation transformation into MSC2 enhances their therapeutic potential for immune and inflammatory diseases via acquiring anti-inflammatory phenotypes before application. Pretreatment of MSCs with the TLR3 agonist, polyinosinic : polycytidylic acid (poly(I : C)) has been found to transform MSCs into the MSC2 phenotype with enhanced anti-inflammatory effects, however, the molecular mechanisms underlying the transition to enhanced anti-inflammatory properties remain unclear.

Acute pancreatitis (AP) is an inflammatory disease characterized by the activation of digestive proteases, inflammatory cells, and necrosis of pancreatic tissue. The severity of pancreatic pathological injuries in acute pancreatitis correlates significantly with the levels of infiltrated inflammatory cell-mediated cytokines, such as IL-1*β*, IL-6, and tumor necrosis factor-alpha (TNF-*α*) [10–12]. Systemic and topical anti-inflammatory approaches are the current strategies for treating pancreatitis [13, 14], and MSCs have shown promise in mitigating pancreatitis severity due to their excellent inflammation-regulating properties [15, 16]. However, their therapeutic effectiveness in clinical settings has not been consistently significant, and improving effectiveness was the major bottleneck problem that limited the clinical application of MSCs. Therefore, this study aims to transform adipose-derived stem cells (ADSCs) into MSC2 phenotype by pretreated with TLR3 agonist poly(I : C), to evaluate the anti-inflammatory properties in a pancreatitis mouse model and to explore the mechanisms of enhanced anti-inflammatory effects.

## 2. Methods

### 2.1. Mice

Male C57BL/6 mice aged 8–10 weeks old were purchased from the Model Animal Research Center of Nanjing University (Nanjing, China). All mice were housed in an SPF standard room under a 12/12 hr light–dark cycle at 24°C, the water and the standard laboratory chow were given ad libitum. All methods or experimental protocols were carried out by The Principles of Laboratory Animal Care and approved by the experimental animal ethics committee of China Pharmaceutical University.

### 2.2. ADSCs Isolation, Culture, and Identification

ADSCs were isolated and cultured from 8–10 weeks old mouse epididymal fat pad by collagenase digestion methods [17]. Briefly, the epididymal fat pad was separated, manually minced into 2–3 mm^2^ pieces, and then digested in HBSS containing with 1 mg/mL of Collagenase I at 37°C for 25 min. The cells were collected by centrifugated at 500 *g*, filtered with 70 *μ*m cell strainer, and resuspended in DMEM/F12 medium (Gibco, New York, USA) supplemented with 10% FBS (Gibco, New York, USA) and 100 mg/mL penicillin/streptomycin. After 48 hr of incubation at 37°C and 5% CO_2_, the unadherent cells were discarded and replenished cell culture medium.

The ADSCs were identified by flow cytometry and multilineage differentiation. The surface marker was detected by flow cytometry (BD FACS Celesta, Becton Dickinson) and analyzed by FACS Diva (BD FACSAria software) after being stained with fluorescein-labeled anti-mouse monoclonal antibodies or mouse IgG isotype control antibodies (BioLegend, California, USA). For multilineage differentiation, the passage three ADSCs were cultured in a differentiation medium. The adipogenesis was carried out in an H-DMEM containing 0.5 mM IMBX, 0.5 *μ*M dexamethasone, 200 *μ*M indomethacin, and 1 *μ*g/mL insulin; the chondrogenesis was carried out in a medium supplemented with 0.1 *μ*M dexamethasone, 10 ng/mL TGF-*β*1, 50 nM ascorbic acid, and 6.25 *μ*g/mL insulin; and the osteogenesis was carried out in medium added with 10 mM *β*-glycerophosphate, 0.1 *µ*M dexamethasone, and 50 *μ*g/mL ascorbic acid. The differentiation potential was assessed via Oil Red O staining, Alixin blue staining, and Alizarin Red staining, respectively.

### 2.3. Pre-Treatment of Poly(I : C)

The passage three ADSCs were treated with poly(I : C; Sigma, Missouri, USA) for 24 hr [4], and the control ADSCs were left untreated. The cell's supernatant or cells were collected for further research.

### 2.4. Cell Transfection

ADSCs were transfected with sh IL-10 lentiviruses or a control lentiviruses (Suzhou Jima Biotechnology Co., Ltd., Suzhou, China). Briefly, the lentivirus plasmids or Lipofectamine 2000 were diluted by Opti-MEM without serum medium according to the manufacturer's recommended optimal concentration. The solutions were gently mixed, followed by a period of keeping them static, before adding the mixtures to the culturing. The transfection rate was identified by immunofluorescence and IL-10 level in ADSCs cultured supernatant.

### 2.5. Cell Migration and Homing in Vivo

The migration assays were performed using a Transwell migration assay as described previously. Briefly, the ADSCs or poly(I : C)-treated ADSCs were plated in 24-well Transwell inserts (12 *μ*m, Corning, New York, USA) at a density of 5 × 10^4^ cells in an upper chamber with 2% FBS, the lower chamber was filled with basic medium supplemented 50 ng/mL CCL2 (Peprotech, New Jersey, USA) as a chemotaxis force. Cells were cultured for another 16 hr after the chemotaxis was added. The cells were fixed with 4% paraformaldehyde, and stained with 0.1% crystal violet. The cells on the underside of the insert were counted under a light microscope (IX53, Olympus, Tokyo, Japan). For ADSCs homing in vivo, the ADSCs were stained with 10 *μ*M 1,1′-dioctadecyl-3,3,3′, 3′-tetramethylindocarbocyanine perchlorate (Dil, Beyotime Biotechnology, Nanjing, China). Cell pellets were adjusted to 2.5 × 10^5^ in 0.1 mL, and administrated to acute pancreatitis mice via tail vein injection in 0.1 mL per mouse. The mice were sacrificed at 24 hr after Dil-labeled ADSCs injection, and the homing capacity was analyzed by immunofluorescence.

### 2.6. Pancreatic Acinar Cell Isolation

The mouse pancreatic acinar cells were separated using the collagenase digestion methods [18]. Briefly, retrograde injection of 1 mg/mL collagenase Ⅴ(Sigma) into the bile duct slowly until the pancreas inflated in completely. The inflated pancreas was dissociated in a 15 mL centrifugal tube containing 1 mg/mL collagenase Ⅴ in a 37°C water bath for 15 min, and filtered through a 70 *μ*m plastic strainer. The suspended cells were centrifugated in 50 g for 2 min, and washed with PBS in twice before cultured in RPMI medium 1640 containing 10% FBS and 100 mg/mL penicillin/streptomycin.

### 2.7. Coculture of Pancreatic Acinar Cells, RAW 264.7 Macrophage, and ADSCs

The pancreatic acinar cells, RAW 264.7 macrophage, cell-to-cell contact cocultured macrophage, and pancreatic acinar cells, were cocultured with ADSCs in a Transwell system. Briefly, the RAW 264.7 cell or pancreatic acinar cells, cocultured macrophages, and pancreatic acinar cells in cell-to-cell contact (the monolayer of pancreatic acinar cells was added into adherent macrophage of 50% confluence) were seeded on the bottom of the plate and the ADSCs were seeded on the six-well Transwell inserts (1.0 *μ*m; Corning) with 80% confluence. Following 6 hr cultured in their respective medium, the ADSCs were cocultured with lower chamber cells with the stimulation of the 50 *μ*M taurocholate (TCI (Shanghai) Development Co., Ltd., Shanghai, China) or not for 24 hr.

### 2.8. Taurocholate-Induced Pancreatitis and ADSCs Treatment

The adaptive mice were randomly divided into four groups (eight mice per group): PBS-treated group (Sham), taurocholate-induced AP group (AP), poly(I : C)-treated ADSCs groups (poly(I : C)-ADSC) and untreated ADSCs groups (Con-ADSC). The mouse model of AP was established by retrograde injection of 5% sodium taurocholate into the pancreatic duct using a microinjection pump [19]. Briefly, the mouse was anesthetized by isoflurane, bile duct was clamped 1 cm from the papilla of Vater by a miniature aorta clamp, and, a small puncture was made through the duodenal wall with a 23-G needle in parallel to the papilla of Vater. A nonradiopaque polyethylene catheter connected to a microinfusion pump (RWD Life Science Co., Ltd., Shenzhen, China) was inserted through the punctured hole in the duodenum and 2 mm into the common bile duct, and then 20 *µ*L of 5% taurocholate or equivolumetric PBS was retrogradely infused over 5 min into the pancreatic duct. After the abdomen was closed in two layers, mice were treated with ADSCs in 2.5 × 10^5^ per mouse or equivolumetric PBS by the tail vein injection, respectively. Twenty-four hours after the taurocholate injection, mice were sacrificed by pentobarbital sodium, and the blood, pancreatic, and lung tissue samples were harvested for subsequent experiments.

### 2.9. Histological Examination

Pancreatic or lung specimens were fixed in 4% paraformaldehyde for 48 hr and then paraffin-embedded and sectioned at a thickness of 5 *μ*m. The tissue sections were deparaffinized and rehydrated sequentially in xylene, xylene/ethanol, gradient ethanol, and distilled water. The tissue sections were then stained with hematoxylin and eosin (H&E) following standard protocols. The images were obtained using fluorescence microscopy (IX53, Olympus, Tokyo, Japan), and two investigators who were blind to the experimental treatment were invited to score the severity of the damage. The histopathological scoring was counted as the average number for 10 fields per slice at magnification 400x. The histopathological scoring of pancreatic injury was shown in Table 1, and the lung histopathological scoring was calculated by a Smith scoring including edema, alveolar or interstitial inflammation, and alveolar or interstitial hemorrhages. The total injury score is calculated as the sum of the scores for respective parameters.

### 2.10. Amylase and Lipase Activity Assay and Cytokines Measurement

The blood samples were obtained from the abdominal aorta, and centrifuged at 1,000 *g* for 5 min at 4°C. The amylase (AMY) activity, lipase activity, and cytokines in serum or cell supernatant were detected by a commercial ELISA Kit (Boshen Biotechnology Co., Ltd., Nanjing, China) according to the manufacturer's instruction.

### 2.11. Immunofluorescence

Paraffin-embedded pancreatic tissue sections were dewaxed using xylene, and rehydrated through gradient alcohol. The sections were microwave reparation in antigen retrieval solution and incubated with mouse F4/80 (duration in 1 : 300), MPO (duration in 1 : 500), CD86 (duration in 1 : 400), and CD206 (duration in 1 : 400) antibodies (Santa Cruz Biotechnology, California, USA) and following incubated with fluorescein-conjugated antibodies (Zhongshan Jinqiao Co., Beijing, China). Images were obtained using a Zeiss LMS700 laser scanning confocal microscope (Zeiss, Oberkochen, Germany) and analyzed using the Image-Pro plus 6.0 image analysis software (Media Cybernetics, Silver Spring, MD, USA).

### 2.12. Cell Apoptosis

The apoptosis of pancreatic acinar cells was detected by a terminal deoxynucleotidyl-transferase-mediated 2′-deoxyuridine 5′-triphosphate nick-end labeling (TUNEL) Assay (Beyotime Biotechnology, Nanjing, China) according to the manufacturer's instructions. The images were photographed using an Olympus IX53 fluorescence microscope (Olympus Corporation, Tokyo, Japan).

### 2.13. PCR

The cells or tissue were harvested and lysed by TRIzol reagent (Takara, Shiga-ken, Japan), and the total RNA was extracted and reverse-transcribed into cDNA using ABM G490 First-strand cDNA Transcription Kit (ABM, shanghai, China). Real-time quantitative PCR was carried out using SYBR Green Master Mix (Toyobo, Osaka, Japan) according to the manufacturer's protocols with the Roche light 480 Real-Time Quantitative PCR employed biosystems (Roche, Basel, Swiss). The gene primer sequences were obtained from General Biol Co. Ltd. (Anhui, China) as shown in Table 2.

### 2.14. Western Blot

Cells or pancreatic tissue were lysed with whole-cell lysis buffer containing phenylmethylsulfonyl fluoride (PMSF), and the total protein was quantified using a Bicinchoninic acid (BCA) kit (Nanjing KeyGEN Biotech. Co. Ltd., Jiangsu, China). Equivalent proteins from each group sample were loaded on 10% sodium dodecyl sulfate–polyacrylamide gel electrophoresis (SDS-PAGE), then transferred onto polyvinylidene fluoride (PVDF) membranes (Merck Millipore Co., Billerica, USA). Blots were blocked with 5% skimmed milk and probed with IKB*α*, IKK*α*, IKK*β*, JAK2, STAT3, Bcl2, Bax, cleaved-caspase3 (Abcam, Cambs, England) primary antibodies at a dilution of 1 : 1,000. The membranes were subsequently incubated with the corresponding HRP-coupled secondary antibodies, and the signals were detected by enhanced chemiluminescence (ECL) HRP-substrate luminol (Merck Millipore Co., Billerica, USA) using a Tanon 5200 Chemiluminescence Apparatus (Shanghai Tanon Life Sciences Co., Ltd.). Quantity One Software was used to quantify the relative protein levels.

### 2.15. Statistical Analysis

Statistical analysis was performed by SPSS 22.0 software. Results are presented as mean ± standard deviation (SD). The continuous data were analyzed by ANOVA; the histological score data were analyzed by a Mann–Whitney rank sum test, and *P* < 0.05 was considered statistically significant.

## 3. Results

### 3.1. Characteristics of ADSCs

**Figure 1:**
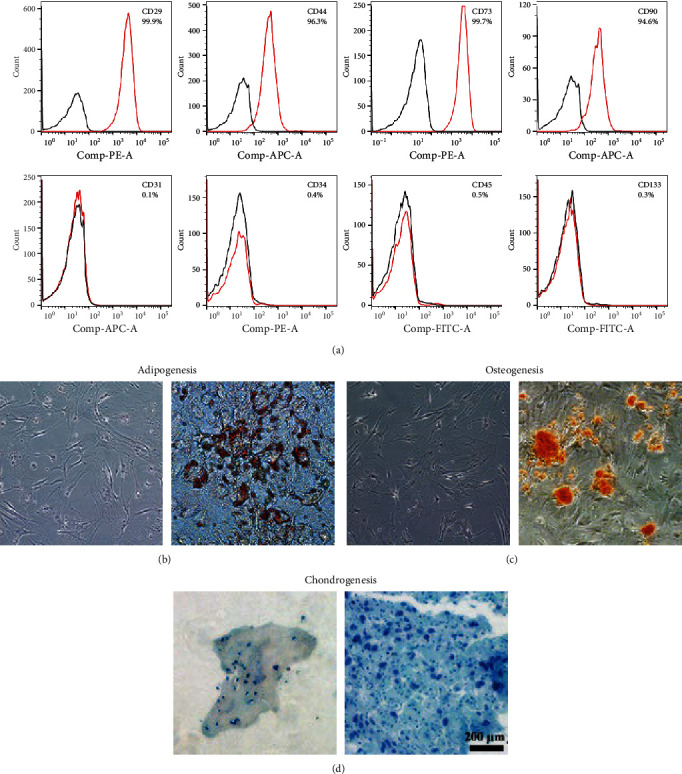
The characteristics of mouse ADSCs: (a) the cell marker of ADSCs detected by flow cytometry (Red and gray lines represent antibodies and their Iso-types, respectively). (b–d) Differentiation of ADSC: (b) adipogenic differentiation potential of ADSCs by Oil Red O staining, (c) osteogenic differentiation potential of ADSCs by Alizarin red S staining, (d) chondrogenic differentiation potential of ADSCs by Alixin blue staining (left: cultured with H-DMEM, right: cultured with differentiation medium; Bar = 200 *μ*m).

Plastic-adherent cells isolated from mouse adipose tissue were successfully propagated. Flow cytometry analysis of passage three cells revealed strong positivity for the surface markers CD29, CD44, CD73, and CD90, while lacking expression of CD31, CD34, CD45, and CD133 (Figure 1(a)). Moreover, these cells exhibited favorable differentiation potential under specific adipogenesis, chondrogenesis, and osteogenesis differentiation media (Figure 1(b)–1(d)).

### 3.2. Poly(I : C) Stimulated ADSCs Result in an Anti-Inflammatory Phenotype

**Figure 2:**
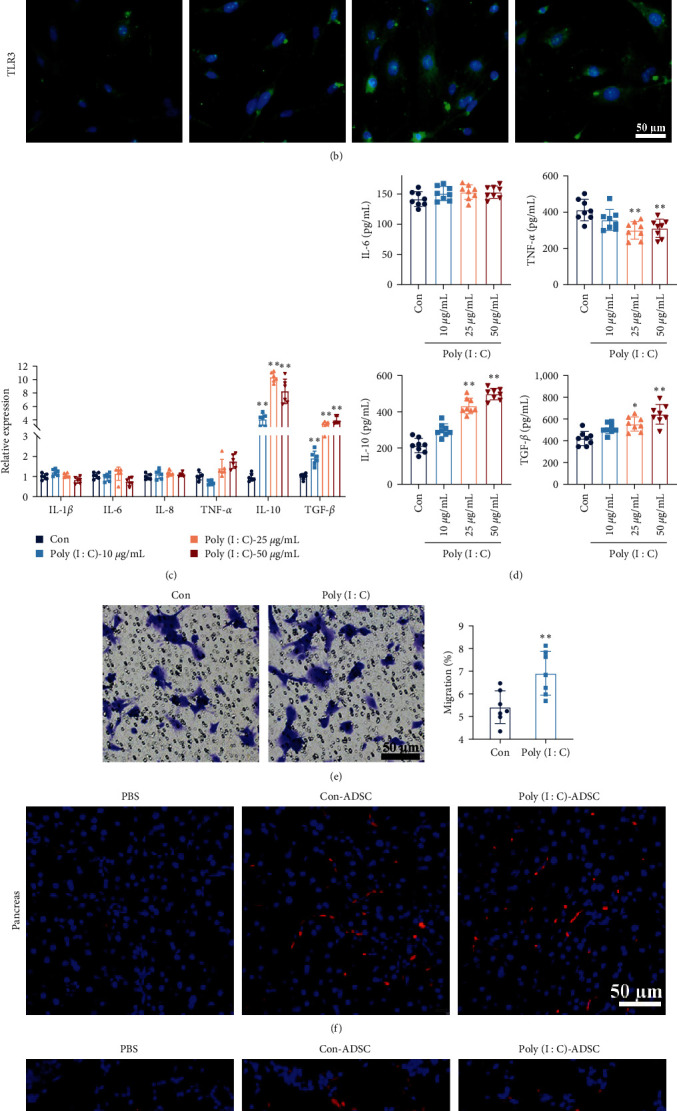
The anti-inflammatory properties of poly(I : C) treated ADSCs: (a) the representative morphology of ADSCs with 10–50 *μ*g/mL poly(I : C) for 24 hr (Bar = 200 *μ*m), (b) the poly(I : C) stimulation activated the expression of TLR3, detected by immunofluorescence (green: TLR3, blue: DAPI to indicate the nucleus; *n* = 3; Bar = 50 *μ*m), (c) the anti-inflammatory or proinflammatory cytokines gene expression with poly(I : C) stimulation (*n* = 6), (d) the levels of IL-6, IL-10, TNF-*α*, and TGF-*β* in poly(I : C) stimulated ADSCs culture supernatant, (e) the migration of 25 *μ*g/mL poly(I : C) treated ADSCs (*n* = 8) and (f, g) The homing capacity of ADSCs in taurocholate-induced pancreatitis mouse pancreas and lung after 24 hr tail vein injection with 2.5 × 10^5^ per mice (red: Dil-labeled ADSCs, blue: DAPI to indicate the nucleus; *n* = 6; Bar = 50 *μ*m). The data were represented as mean ± SD, the post hoc Tukey correction was performed after ANOVA was used for comparison of more than two groups.  ^*∗*^*P* < 0.05,  ^*∗∗*^*P* < 0.01.

The immunomodulation capability of MSCs has been shown primarily through paracrine signaling and TLR3 activation, leading to altered secretomes. Immunofluorescence results revealed that treatment with 10–50 *μ*g/mL poly(I : C) elevated the expression of TLR3 in ADSCs without any significant morphological changes (Figures 2(a) and 2(b)). Paracrine measurements indicated an increase in both the expression and secretion of anti-inflammatory cytokines, such as IL-10 and TGF-*β*, in poly(I : C)-treated ADSCs, while the expression and secretion of proinflammatory cytokines were decreased (Figures 2(c) and 2(d)), particularly in ADSCs simulated with 25 and 50 *μ*g/mL poly(I : C). However, no significant difference was observed between ADSCs treated with 25 and 50 *μ*g/mL poly(I : C). Consequently, ADSCs were stimulated with 25 *μ*g/mL poly(I : C) in subsequent experiments quickly homing to inflammatory tissue is the important factor for MSCs perform anti-inflammatory efficacy, and this process is regulated by TLR3 activation. The migration assay demonstrated that poly(I : C)-treated ADSCs exhibited strengthened migration capabilities under the chemotaxis force of CCL2 (Figure 2(e)). However, enhanced homing capability was not observed in the pancreas and lung of mice with pancreatitis (Figures 2(f) and 2(g)). These results indicate that TLR3 activation promotes the transformation of ADSCs into an anti-inflammatory phenotype, potentially with strengthened properties in inhibiting inflammation.

### 3.3. Poly(I : C) Treated ADSCs Amplify the Therapeutic Capability in Acute Pancreatitis

**Figure 3:**
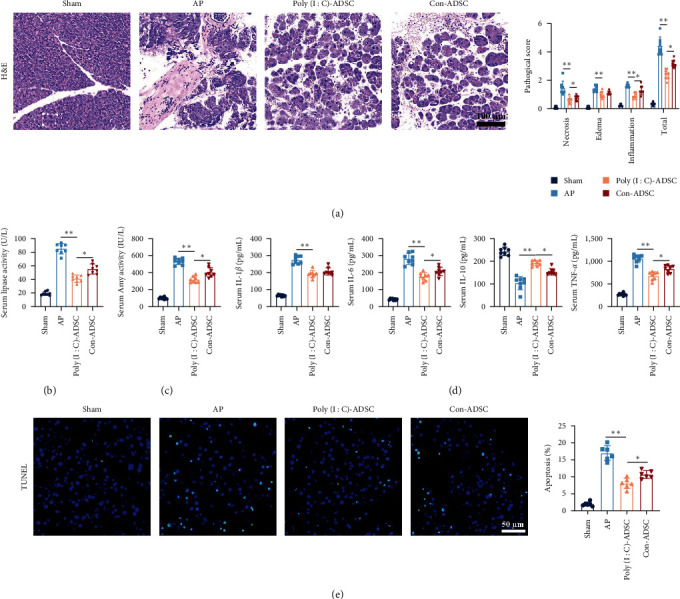
Poly(I : C) stimulation amplifies the anti-inflammatory capability of ADSCs in taurocholate-induced pancreatitis: (a) the representative histopathologic images of mouse pancreas and pathology scores. H&E staining (*n* = 8, bar = 100 *μ*L), (b) amylase activity in mouse serum (*n* = 8), (c) lipase activity in mouse serum (*n* = 8), (d) the levels of IL-1*β*, IL-6, IL-10, and TNF-*α* in mouse serum (*n* = 8), (e) the representative images of TUNEL staining of the pancreas (green: TUNEL positive cell, blue: DAPI to indicate the nucleus; *n* = 6, bar = 50 *μ*m). The data were represented as mean ± SD, the post hoc Tukey correction was performed after ANOVA was used for the comparison of more than two groups.  ^*∗*^*P* < 0.05,  ^*∗∗*^*P* < 0.01.

The enhanced anti-inflammatory properties of poly(I : C)-treated ADSCs were evaluated in taurocholate-induced pancreatitis mice. Initial examination of pancreatic histopathological damage revealed significant mitigation of edema, necrosis, and inflammation in the pancreas of mice treated with poly(I : C)-ADSCs compared with untreated ADSCs (Figure 3(a)). The activities of amylase and lipase were both further decreased in the serum of mice treated with poly(I : C)-ADSCs (Figures 3(b) and 3(c)). Additionally, serum levels of IL-1*β*, IL-6, and TNF-*α* decreased, while the level of IL-10 increased in poly(I : C)-treated ADSCs compared with untreated ADSCs (Figure 3(d)). Similarly, apoptosis, detected by TUNEL stain, also indicated that poly(I : C)-treated ADSCs had favorable effects in inhibiting acinar cell apoptosis (Figure 3(e)). These results suggest that poly(I : C)-treated ADSCs exhibit strengthened anti-inflammatory effects compared with untreated ADSCs in mice.

### 3.4. Poly(I : C)-Treated ADSCs with Strengthened Effects in Pancreatitis-Associated Lung Injury

**Figure 4:**
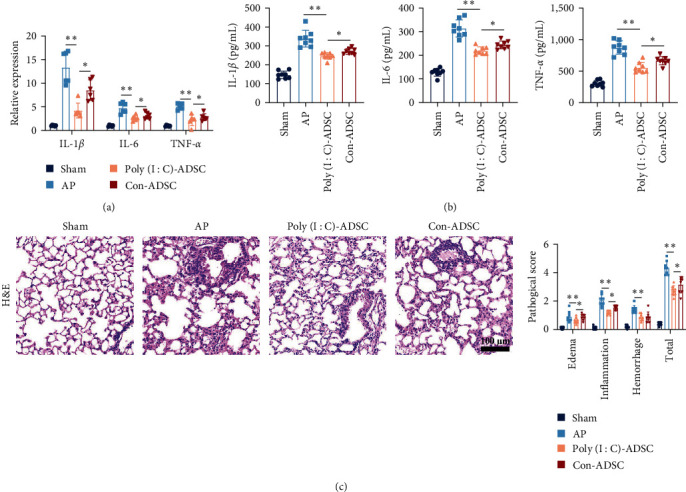
Poly(I : C) treated ADSCs with enhanced anti-inflammatory effects in pancreatitis-associated lung injury: (a) the mRNA expression of IL-1*β*, IL-6, and TNF-*α* in mouse lung tissue (*n* = 6), (b) the cytokines of IL-1*β*, IL-6, and TNF-*α* in lung tissue (*n* = 8), and (c) the representative histopathologic images of mouse lung and pathology scores (*n* = 6, bar = 100 *μ*m). Data were represented as mean ± SD, the post hoc Tukey correction was performed after ANOVA was used for comparison of more than two groups.  ^*∗*^*P* < 0.05,  ^*∗∗*^*P* < 0.01.

Given that lung injury is a common and severe complication associated with mortality in pancreatitis, the impact of poly(I : C)-treated ADSCs on lung tissue inflammation was assessed. Results showed that poly(I : C)-treated ADSCs exhibited enhanced effects in suppressing proinflammatory cytokine gene expression and cytokine levels in the lung tissue of pancreatitis mice (Figures 4(a) and 4(b)). Histopathological examination revealed strengthened inhibitory effects on lung injury, with reduced severity of leakage, hemorrhage, and inflammatory cell infiltration in mice administered with poly(I : C)-treated ADSCs (Figure 4(c)).

### 3.5. Poly(I : C)-Treated ADSCs Strengthened Their Capability to Inhibit Inflammatory Signaling

**Figure 5:**
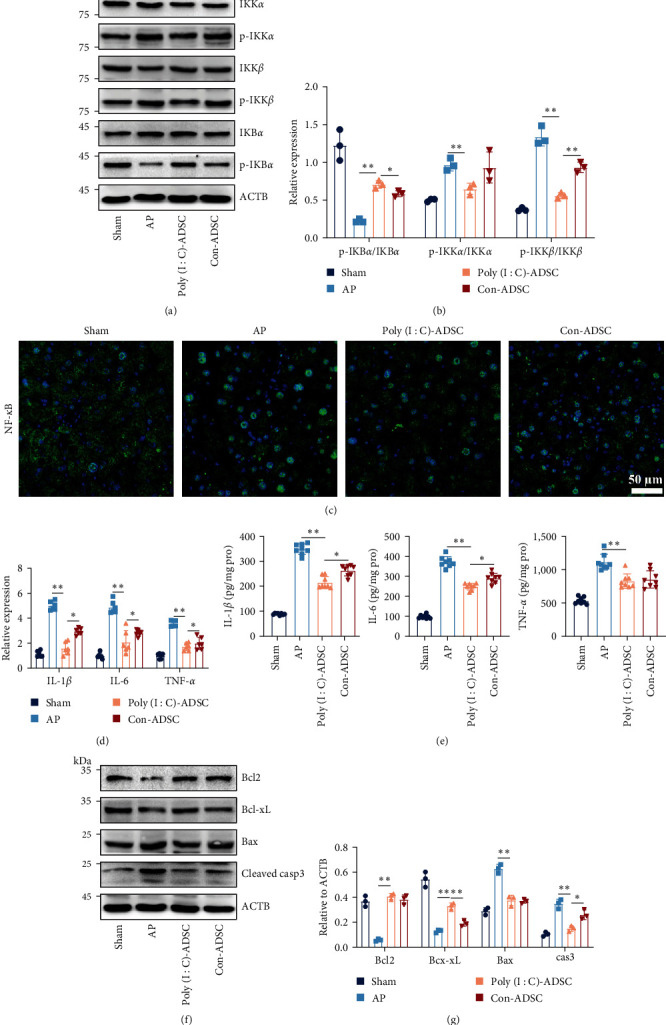
Poly(I : C) treated ADSCs with favorable effects in NF-*κ*B signaling transduction in taurocholate-induced pancreatitis mice: (a, b) the protein expression of IKB and IKK*α*/*β* in the pancreas (*n* = 3), (c) nuclear translocation of NF-*κ*B in ADSCs treatment pancreatitis mouse pancreas (*n* = 6, Bar = 50 *μ*m), (d) the proinflammatory cytokines gene expression of IL-1*β*, IL-6, and TNF-*α* in the pancreas (*n* = 6), (e) the levels of IL-1*β*, IL-6, and TNF-*α* in pancreas tissue (*n* = 8), and (f, g) the protein expression of Bax, cleaved-caspase 3, Bcl2, and Bcl-xL in the pancreas (*n* = 3). Data were represented as mean ± SD, the post hoc Tukey correction was performed after ANOVA was used for comparison of more than two groups.  ^*∗*^*P* < 0.05,  ^*∗∗*^*P* < 0.01.

To elucidate the mechanism underlying the strengthened anti-inflammatory effects of poly(I : C)-treated ADSCs, we focused on the NF-*κ*B signaling that plays role in the initiation and cascade of inflammation. Results revealed a decrease in IKB degradation and IKK*α*/*β* phosphorylation in pancreatic tissues of mice treated with poly(I : C)-treated ADSCs compared with untreated ADSCs (Figures 5(a) and 5(b)). Nuclear translocation of NF-*κ*B, which crucial for cytokine production initiation, was also significantly reduced in poly(I : C)-treated ADSCs (Figure 5(c)), leading to decreased expression and protein levels of proinflammatory cytokines, including IL-1*β*, IL-6, and TNF-*α* (Figures 5(d) and 5(e)). Furthermore, poly(I : C)-treated ADSCs exhibited a favorable inhibitory effect on the expression of apoptosis-associated proteins, such as caspase 3 and Bax (Figures 5(f) and 5(g)). These results indicate that the enhanced anti-inflammatory effect of poly(I : C)-treated ADSCs is associated with their robust inhibition on NF-*κ*B signaling.

### 3.6. Favorable Anti-Inflammatory Effects of Poly(I : C)-Treated ADSCs Associated with Macrophages

**Figure 6:**
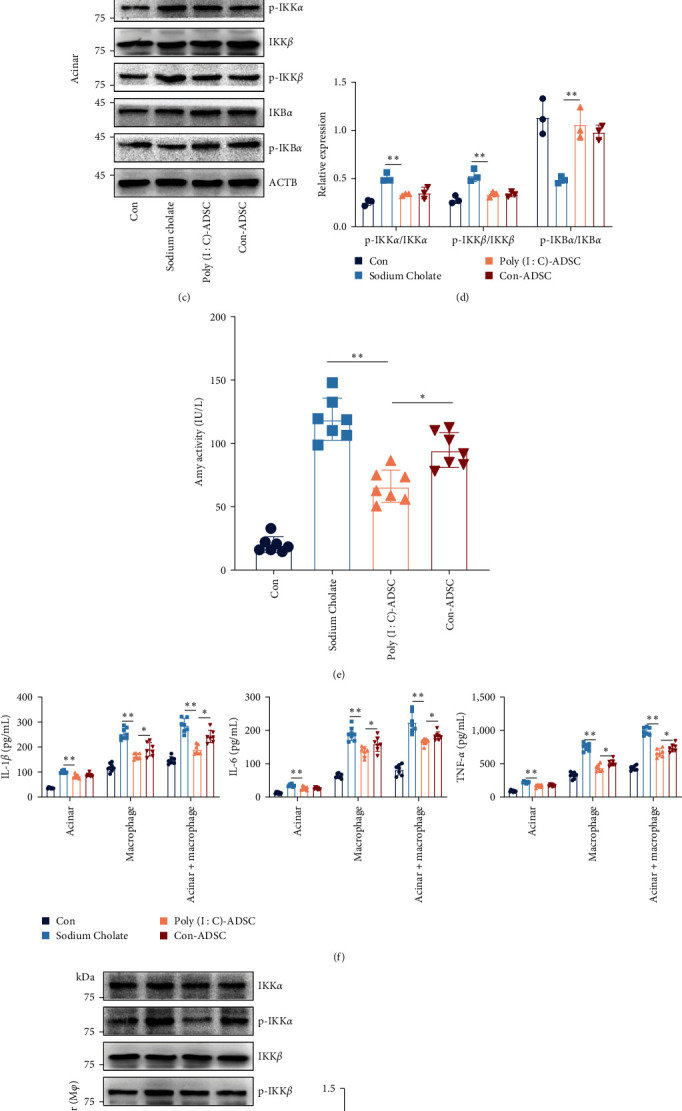
The favorable anti-inflammatory effects of poly(I : C) treated ADSCs on NF-*κ*B transduction depended on regulating macrophage: (a) diagram of the model for coculture of ADSCs and pancreatic acinar cell or macrophage, (b) the amylase activity in cocultured acinar cells (*n* = 8), (c, d) the protein expression of IKB and IKK*α*/*β* in cocultured acinar cells (*n* = 3), (e) the amylase activity in the acinar cell cocultured with macrophage in cell-to-cell contract (*n* = 8), (f) the levels of IL-1*β*, IL-6, and TNF-*α* in supernatant from the lower chamber in different cocultured models (*n* = 8) and (g, h) the protein expression of IKB and IKK*α*/*β* in acinar cells from pancreatic acinar cells cocultured with macrophage in cell-to-cell contract (*n* = 3). The data were represented as mean ± SD, the post hoc Tukey correction was performed after ANOVA was used for the comparison of more than two groups.  ^*∗*^*P* < 0.05,  ^*∗∗*^*P* < 0.01.

We assessed whether the favorable anti-inflammatory effects of poly(I : C)-treated ADSCs were associated with macrophages using a Transwell coculture system in further (Figure 6(a)). Results showed that coculturing with ADSCs suppressed amylase activity, but poly(I : C)-treated ADSCs did not exhibit strengthened capability in suppressing amylase activity in cocultured acinar (Figure 6(b)). Notably, poly(I : C)-treated ADSCs demonstrated a favorable capability in suppressing proinflammatory cytokine production and inhibiting NF-*κ*B activation in vivo. However, there were no differences observed in IKB*α* degradation and IKK*α*/*β* phosphorylation between poly(I : C)-treated and untreated ADSCs in cocultured acinar cell (Figures 6(c) and 6(d)). These findings led us to speculate that other mechanisms contribute to the therapeutic efficacy of poly(I : C)-treated ADSCs in pancreatitis. Cytokine interactions between acinar cells and inflammatory cells, especially macrophages, are critical early events in the initiation and progression of pancreatitis. To investigate this, RAW264.7 macrophages were cocultured with pancreatic acinar cells in cell-to-cell contact in the lower chamber and cocultured with ADSCs in the insert using a Transwell system (Figure 6(a)). The results showed a sharp increase in amylase activity when cocultured in cell-to-cell contact, whereas poly(I : C)-treated ADSCs exhibited strengthened inhibition of amylase activity in the culture supernatant (Figure 6(e)). These findings suggest signaling communications between pancreatic acinar cells and macrophages, where macrophage infiltration promotes pancreatic acinar cell damage through communications. The favorable inhibitory effects of poly(I : C)-treated ADSCs on acinar cell damage may also be associated with the regulation of macrophages. To further explore this, cytokines in macrophages cocultured with pancreatic acinar cells in cell-to-cell contact by Transwell coculture system were evaluated. The results showed that poly(I : C)-treated ADSCs had stronger effects in decreasing the proinflammatory cytokine level in supernatant of lower chamber, such as IL-1*β*, IL-6, and TNF-*α* (Figure 6(f)). Consistently, acinar cells cocultured with macrophages in cell-to-cell contact also exhibited a decreasing of NF-*κ*B signaling activation in poly(I : C)-treated ADSCs (Figures 6(g) and 6(h)). These results indicate that the favorable effects of poly(I : C)-treated ADSCs in suppressing inflammation in pancreatitis were critically associated with the regulation of macrophage inflammation.

### 3.7. Poly(I : C)-Treated ADSCs with Potent Effects in Macrophage Polarization

**Figure 7:**
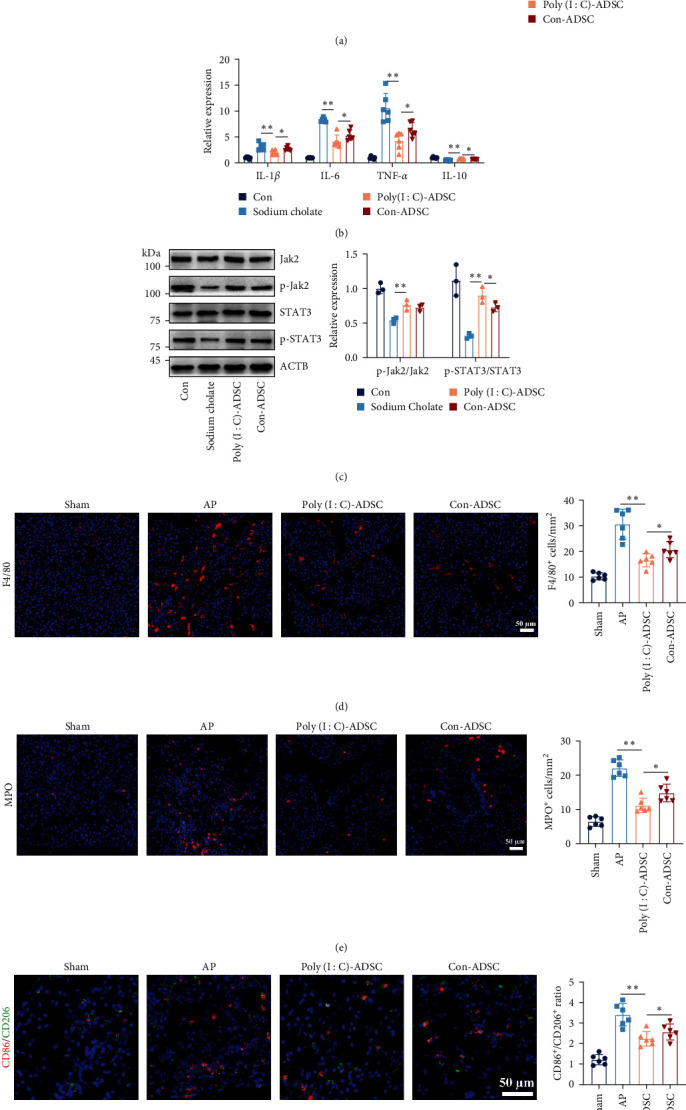
The poly(I : C) treated ADSCs with superior effects in skewing macrophage into anti-inflammatory phenotype: (a) the classically or alternatively activated macrophages were representative of CD86 and CD206 using a flow cytometry assay (*n* = 3), (b) the mRNA expression of IL-1*β*, IL-6, TNF-*α*, and IL-10 in cocultured macrophage (*n* = 6), (c) the protein expression of JAK2 and STAT3 in cocultured macrophage (*n* = 3), (d) the infiltration of macrophage (F4/80), (e) neutrophils (MPO; red: F4/80 or MPO positive cell, blue: DAPI to indicate the nucleus), and (f) polarization of macrophage (red: CD86 positive cell, green: CD206 positive cell, blue: DAPI to indicate the nucleus) in ADSCs treatment pancreatitis mouse pancreas (*n* = 6, Bar = 50 *μ*m). The data were represented as mean ± SD, the post hoc Tukey correction was performed after ANOVA was used for the comparison of more than two groups.  ^*∗*^*P* < 0.05,  ^*∗∗*^*P* < 0.01.

Given that proinflammatory cytokines are mainly produced by classically activated macrophages, we evaluated whether the strengthened effects of poly(I : C)-treated ADSCs in decreasing pancreatic acinar cell damage are associated with macrophage polarization. Results from a coculture system demonstrated that poly(I : C)-treated ADSCs had a favorable effect in inhibiting classically activated macrophages induced by taurocholate, as evidenced by decreased expression of classically activated macrophage characteristic genes (Figures 7(a) and 7(b)). Additionally, poly(I : C)-treated ADSCs cocultured with macrophages exhibited increased phosphorylated levels of Jak2 and STAT3, transcription factors involved in alternative macrophage activation (Figure 7(c)). Evaluation of neutrophils and macrophage infiltration revealed significantly decreased numbers in the pancreas of mice administered poly(I : C)-treated ADSCs (Figure 7(d) and 7(e)). Moreover, poly(I : C)-treated ADSCs administration resulted in a higher number of CD206 positive macrophages, indicative of an alternative activation state (Figure 7(f)). These findings demonstrate that poly(I : C)-treated ADSCs exhibit potent effects in inhibiting macrophage inflammation, which is associated with regulating macrophage classical/alternative transformation and mitigating macrophage cytokine-mediated acinar damage.

### 3.8. Strengthened Anti-Inflammatory Property of Poly(I : C)-Treated ADSCs via IL-10 Secretion

**Figure 8:**
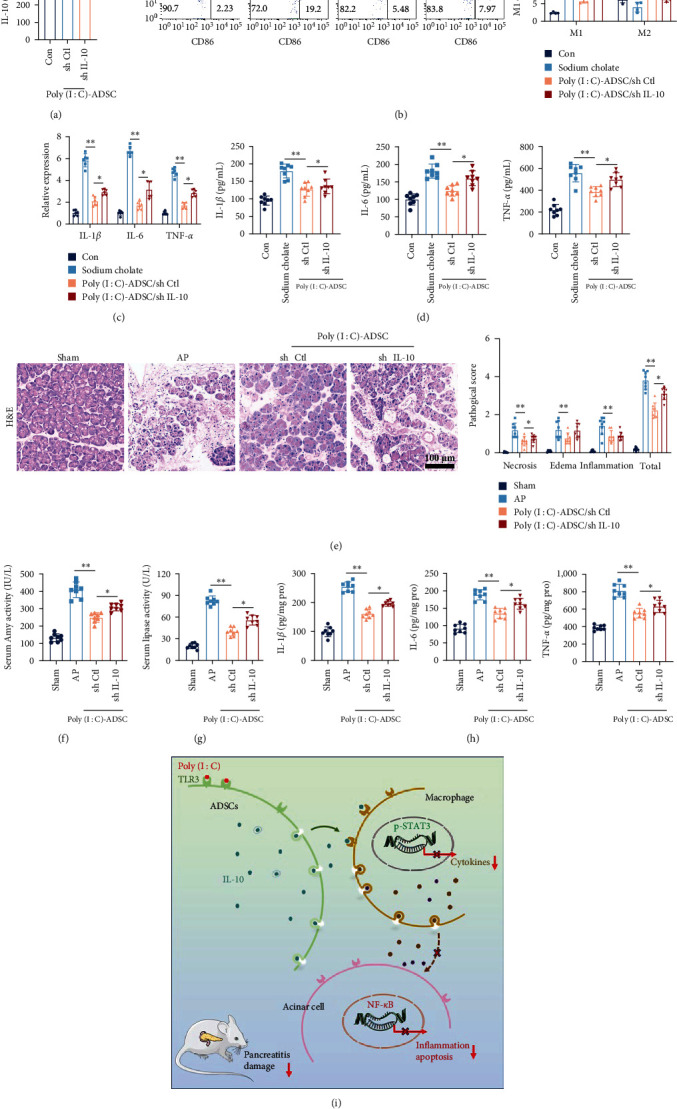
Poly(I : C) treated ADSCs with strengthened anti-inflammatory effects via IL-10: (a) the level of IL-10 in the supernatant of IL-10 knocked down Poly(I : C)-treated ADSCs (*n* = 8), (b) the classically or alternatively activated macrophages were representative of CD86 and CD206 using a flow cytometry assay (*n* = 3), (c) the mRNA expression of IL-1*β*, IL-6, and TNF-*α* in macrophages that cocultured with IL-10 knocked down Poly(I : C)-treated ADSCs (*n* = 6) (d) the levels of IL-1*β*, IL-6, and TNF-*α* in macrophages that cocultured with IL-10 knocked down Poly(I : C)-treated ADSCs (*n* = 8), and (e) the representative histopathologic images of mouse pancreas and pathology scores of IL-10 knocked down Poly(I : C)-treated ADSCs treated mice. H&E staining (*n* = 8, Bar = 100 *μ*L), (f, g) Serum amylase activity and lipase activity of IL-10 knocked down Poly(I : C)-treated ADSCs treated mice (*n* = 8), (h) the levels of cytokines of IL-10 knocked down Poly(I : C)-treated ADSCs treated mice pancreatic tissue (*n* = 8) and (i) graphical abstract: Poly(I : C)-mediated activation of TLR3 contributes to increased secretion of IL-10 in ADSCs, and strengthens their inflammatory inhibitory on macrophages and acinar cells, thereby exhibiting strengthened property in reducing pancreatitis-induced tissue damage. The data were represented as mean ± SD, the post hoc Tukey correction was performed after ANOVA was used for the comparison of more than two groups.  ^*∗*^*P* < 0.05,  ^*∗∗*^*P* < 0.01.

Given the in vitro results demonstrating higher IL-10 secretion by poly(I : C)-treated ADSCs, we further investigated whether the enhanced anti-inflammatory properties of poly(I : C)-treated ADSCs were associated with increased IL-10 secretion. Poly(I : C)-treated ADSCs were transfected with IL-10 (sh IL-10) or negative control (sh Ctl) lentiviruses, resulting in an obvious reduction of IL-10 in the supernatant of sh IL-10 transfected cells (Figure 8(a)). Transwell coculture experiments with macrophages indicated that knocking down IL-10 impaired the effects of poly(I:C)-treated ADSCs on macrophage classical/alternative transformation (Figure 8(b)). The expression and secretion of inflammatory cytokines in cocultured macrophages followed a similar trend, with IL-10 silencing diminishing the inhibitory effects of poly(I : C)-treated ADSCs on cytokine production (Figures 8(c) and 8(d)). Therefore, the IL-10-mediated anti-inflammatory effects of poly(I : C)-treated ADSCs were further assessed in a pancreatitis mouse model. Results showed that IL-10 knocked down poly(I : C)-treated ADSCs, while still mitigating pancreas damage, exhibited weakened effectiveness compared with negative control knocked down poly(I : C)-treated ADSCs (Figure 8(e)). Amylase and lipase activities followed a similar trend (Figure 8(f) and 8(g)). Finally, the levels of inflammatory cytokines in pancreatic tissues were detected, revealing elevated levels in IL-10 knocked down poly(I : C)-treated ADSCs compared with negative control poly(I : C)-treated ADSCs. However, the IL-10 knocked down poly(I : C)-treated ADSCs still exhibited an inhibitory effect on inflammatory cytokines (Figure 8(h)). These results demonstrate that IL-10 silencing attenuates the inhibitory effect of poly(I : C)-treated ADSCs on inflammation. The strengthened anti-inflammatory property of TLR3-activated ADSCs is associated with IL-10 secretion.

## 4. Discussion

In this study, we chose a mouse model of pancreatitis induced by taurocholate to evaluate the anti-inflammatory properties of ADSCs activated with a TLR3 agonist poly(I : C), and explored the underlying of enhanced anti-inflammatory mechanism. The results demonstrated that poly(I : C) pretreated ADSCs exhibited a predominant anti-inflammatory effect in taurocholate-induced pancreatic inflammation compared with untreated ADSCs. The pancreas damage, pancreatitis-associated lung injury, pancreatic acinar apoptosis, systemic inflammation, and inflammatory cascade were mitigated significantly in poly(I : C) pretreated ADSCs administration mice than untreated ADSCs. Mechanistically, poly(I : C)-treated ADSCs transformed an anti-inflammatory phenotype, characterized by an increased level of IL-10 secretion and with a strengthened immunoregulatory effect (Figure 8(i)). The enhanced immunomodulatory property of poly(I : C) pretreated ADSCs in pancreatitis is not only associated with their elevated levels of anti-inflammatory factors, but also associated with modulating the inflammatory state of macrophages in indirectly (Figure 8(i)). We propose that inducing a specific anti-inflammatory phenotype by TLR3 agonist poly(I:C) in MSCs is a meaningful strategy for improving the therapeutic efficacy of them in inflammatory diseases.

The infiltration of macrophages and neutrophils is a key pathogenic factor contributing to increased secretion of amylase and lipase in acinar cells during acute pancreatitis [20]. The cytokine cascade between pancreatic acinar cells and macrophages is a critical process leading to acinar cell cytoplasmic vacuolization and death, which closely depends on NF-*κ*B signaling activation in both acinar cells and macrophages [21]. Studies have highlighted the crucial role of NF-*κ*B activation in pancreatic acinar cells and the subsequent expression of IL-1*β*, IL-6, and TNF-*α* in the initiation and aggravation of acute pancreatitis through the recruitment of inflammatory cells [22, 23]. This sets off a vicious circle where proinflammatory cytokines produced by infiltrating inflammatory cells, especially neutrophils and macrophages, further damage pancreatic acinar cells, amplifying pancreatic injury [21]. Proinflammatory cytokines are primarily derived from classically activated macrophages and depend on the activation of NF-*κ*B, while alternatively activated macrophages secrete anti-inflammatory cytokines and contribute to tissue regeneration. Therefore, focusing on macrophage polarization is an important therapeutic strategy for treating pancreatitis, and recent studies have indicated that MSCs hold potential for pancreatitis treatment by suppressing NF-*κ*B activation and reducing the expression of proinflammatory cytokines. Despite the potent immunomodulatory and inflammatory suppressive effects of MSCs, improving their clinical effectiveness remains a significant challenge limiting their widespread use. Research has shown that MSCs express several TLRs, affecting their migration, invitation, and secretion capabilities in response to specific TLR agonists [7]. Recently some of the work on the downstream consequences of TLRs provides emerging evidence for a new anti-inflammatory or proinflammatory immune modulating role for MSCs. The specific TLRs result in MSCs with opposite immunomodulatory properties through transformation into distinct phenotypes. Waterman et al. [5] demonstrated that MSCs could transform into two distinct phenotypes following difference TLR stimulation: TLR4 agonists induced a proinflammatory MSC1 phenotype, while TLR3 agonist skewed MSCs into an anti-inflammatory MSC2 phenotype. Further research revealed that MSC2 adopted an immune-suppressive phenotype by secreting anti-inflammatory soluble factors, including IDO, IL-10, PGE2, and TGF-*β* [24]. Conversely, MSC1 exhibited a phenotype characterized by high levels of CCL3, CCL4, CCL9, CCL10, IL-1*β*, and TNF-*α* and low levels of IDO [25]. These findings suggest that the concept of MSCs polarization into an anti-inflammatory phenotype through distinct TLR agonists is an attractive strategy to enhance the anti-inflammatory capabilities of MSCs, and some studies have demonstrated poly(I:C)-pretreated MSCs exhibit strengthened effects in decreasing kidney ischemia/reperfusion injury [26]. However, the situation may be more complex within the disease microenvironment, high concentrations of pro-inflammatory cytokines or low levels of anti-inflammatory cytokinesis trigger MSCs to an immune-suppressive features (MSC2), and secrete high amounts of IDO, TGF-*β*, and IL-10. By contrast, MSCs acquire an immune-activation phenotype (MSC1) under low inflammatory conditions, persistently low and comparable levels of proinflammatory and anti-inflammatory cytokines in the microenvironment lead to the production of low levels of IDO and proinflammatory cytokines, respectively. Therefore, in the application of inflammatory diseases, pretreating MSCs in advance to acquire a strengthened anti-inflammatory property is a meaningful strategy to enhance their clinical effectiveness. Poly (I : C), a synthetic double-stranded RNA (dsRNA) analog, is a molecular pattern associated with viral infection, Poly (I : C) can recognized by TLR3 and acts as the TLR3 agonist in TLR3 positive expression cells. In vitro experiments, Poly (I : C) can bind to the cellular surface TLR3 domain and then activate signaling downstream of TLR3 and regulate the immune process, but also promote TLR3 expression to enhance the cell response to external stimulation. MSCs have been found positive in the vast majority of TLRs, suggesting that MSCs can respond to poly (I : C) stimulation and alter their immune phenotype. Waterman et al. [5] demonstrated that MSCs pretreated with the TLR3 agonist poly (I : C) exhibited enhanced anti-inflammatory properties, in our experiment, poly(I : C) pretreated ADSCs adapted an anti-inflammatory phenotype with higher levels of IL-10 and TGF-*β* secretion, demonstrating a strengthened capability in inhibiting inflammatory cascades in pancreatitis compared with untreated ADSCs. However, the enhanced anti-inflammatory effect was observed only in cocultured macrophages and in cell-to-cell contact cocultured macrophages and acinar cells, but not in cocultured acinar cells alone. These results suggest that the dominant anti-inflammatory effect of poly(I : C) pretreated ADSCs is more likely associated with regulating macrophage inflammation.

In addition to alterations in their metabolic profile, the anti-inflammatory effects of MSCs with an anti-inflammatory phenotype are closely associated with the regulation of inflammation in other immune cells. It is well established that MSCs exhibit potent immunoregulatory effects in both the innate and adaptive immune systems through secretomes or cell-to-cell contacts [27]. The metabolic reprograming of MSCs on macrophages is a key process for their immunomodulatory effects on the innate immune system. Research has shown that MSCs actively interact with components of macrophages, influencing their subsequent inflammatory behavior [28, 29]. Coculture of monocytes with human or mouse MSCs promotes alternatively activated macrophages, and this process is strengthened by overexpression of TSG6 or IDO [30, 31]. Moreover, activation of MSCs with specific TLRs increases the expression of IL-10 and IDO in MSCs, thereby enhancing their capability to promote macrophage alternatively activation and maintain microenvironment homeostasis [32, 33]. In this study, poly(I : C) pretreated ADSCs with the enhanced IL-10 secretion and with strengthen property in regulates macrophage alternatively activation, knocking dowm IL-10 imparied the effectiveness of poly(I : C) pretreated ADSCs on macrophage alternatively activation and cytokines production.

IL-10 is an immunosuppressive cytokine with the ability to suppress cell-mediated and antibody-mediated responses, and it is considered a potential therapy for various inflammatory diseases [34]. Previous studies have shown that IL-10 can suppress the immune response by decreasing cell surface expression of MHC class II and down-regulating the expression of other pro-inflammatory cytokines, such as IL-1*β*, IL-6, and TNF-*α* [35]. Beyond direct immune regulation, IL-10 also affects immune inflammation by regulating the inflammatory metabolism of other immune cells, such as the classically/alternatively activation of macrophages via the phosphorylation of Jak and the nuclear transposition of Stat3 [36, 37]. Consistent with these findings, our results also demonstrated that poly(I : C) pretreated ADSCs exhibit a predominant property in the regulation of macrophage inflammation. Compared with untreated ADSCs, poly(I : C)-treated ADSCs exhibited pronounced phosphorylation of STAT3 in macrophages simultaneously. Importantly, knocking down IL-10 impaired the ability of poly(I : C)-treated ADSCs to inhibit macrophage inflammation and pancreatic damage in mice.

In summary, our results demonstrate an enhanced anti-inflammatory function of poly(I : C) treated ADSCs in taurocholate-induced pancreatitis. The concept of transforming MSCs into an anti-inflammatory phenotype emerges as an attractive strategy to improve the efficiency of MSCs in inflammatory diseases. Pretreatment of MSCs with appropriate concentrations of Poly(I : C) would be an effective means to improve the clinical anti-inflammatory efficacy of MSCs.

## Figures and Tables

**Table 1:** Pathological score of pancreas.

Score	Diffuse expansion edema	Acinar Necrosis (cells/HPF)	Inflammation (leukocytes/HPF)
0	Absent	Absent	0–5
1	Interlobar septae	1–4	6–15
2	Interlobubar septae	5–10	16–25
3	Interacinar septae	11–16	26–35
4	Intercellular spaces	>16	>35

**Table 2:** Primer information.

Genes	Forward	Reverse
*IL-1β*	TGGACCTTCCAGGATGAGGACA	GTTCATCTCGGAGCCTGTAGTG
*IL-6*	TACCACTTCACAAGTCGGAGGC	CTGCAAGTGCATCATCGTTGTTC
*IL-8*	TGCATGGACAGTCATCCACC	ATGACAGACCACAGAACGGC
*IL-10*	CGGGAAGACAATAACTGCACCC	CGGTTAGCAGTATGTTGTCCAGC
*TNF-α*	GGTGCCTATGTCTCAGCCTCTT	GCCATAGAACTGATGAGAGGGAG
*TGF-β*	CGTGGAAATCAACGCTCCAC	GAAGTTGGCATGGTAGCCCT
*ACTB*	CATTGCTGACAGGATGCAGAAGG	TGCTGGAAGGTGGACAGTGAGG

## Data Availability

The data that support the findings of this study are available from the corresponding author, (Liang Jin), upon reasonable request.
